# Early Stage W.H.O. Grade I and II Follicular Lymphoma Treated with Radiation Therapy Alone

**DOI:** 10.1371/journal.pone.0065156

**Published:** 2013-06-06

**Authors:** Naseer Ahmed, Timothy E. Owen, Morel Rubinger, Gaynor Williams, Zoann Nugent, Shahida Ahmed, Andrew Cooke

**Affiliations:** 1 University of Manitoba, Winnipeg, Manitoba, Canada; 2 CancerCare Manitoba, Winnipeg, Manitoba, Canada; 3 Queens University, Kingston, Ontario, Canada; 4 Cancer Centre of Southeastern Ontario, Kingston, Ontario, Canada; University of North Carolina at Chapel Hill, United States of America

## Abstract

**Objectives:**

This retrospective study was undertaken to evaluate the outcome of patients with stage I or II (limited stage), grade I–II follicular non-Hodgkin’s lymphoma (FL) treated with radiation therapy (RT) alone as initial management.

**Methods:**

Patients with stage I or II and pathologically confirmed WHO grade I or II FL treated initially with RT alone between 1982 and 2008 were identified from a population based cancer registry.

**Results:**

Forty patients with a mean age 61.3 years at diagnosis were identified. The median follow up was 6.9 years from the end of radiation therapy. Stage was I (n = 26) and II (n = 14). None had B symptoms. The Follicular Lymphoma International Prognostic Index (FLIPI) was low risk in 26 patients and intermediate risk in 5. Doses ranged from 15 Gy to 48 Gy, with a median dose of 35 Gy. All patients achieved a complete clinical response (CR). 5 and 10 year overall survival (OS) was 86% and 59%, progression free survival (PFS) 67% and 54%. Age ≥60 at diagnosis was associated with reduced OS, *p* = 0.029, but did not affect PFS. No other clinical features including grade or FLIPI were significant for outcomes. Local failure was uncommon occurring in 8% (3/40) although this was 21% (3/14) of all recurrences.

**Conclusions:**

OS and PFS outcomes for radiation alone in limited stage low grade FL patients from this single institution study are consistent with previously published data. No predictors were prognostic for PFS. A dose of ≤35 Gy may be appropriate. In this highly selected homogeneous group the FLIPI loses discriminating ability. Local control is excellent, and a majority of patients are free of disease after 5 years.

## Introduction

Grade I and II follicular lymphoma (FL) account for an estimated 9.5% and 6.2% respectively of all non-Hodgkin’s lymphoma and about 70% of follicular lymphoma [1]. Follicular lymphoma grade described by the World Health Organization (WHO) [2] depends on the number of centroblasts per high power field. Current recommendations [3] are to treat limited stage FL with radiation but the slowly progressive nature of the disease allows treatment to be deferred until long after diagnosis and it is not known if the local control that can be achieved by radiation actually alters overall outcome [4], [5]. A study of larger *vs.* smaller RT volumes showed better freedom from relapse but no difference in survival [6]. The long term follow up required and competing mortality in this generally older population makes determination of cure elusive.

We undertook this study to determine if our own results with radiation alone in limited stage, low grade FL were consistent with published rates of local control, progression free survival (PFS) and overall survival (OS) and to examine possible prognostic factors.

## Methods

### Ethics Statement

RESEARCH ETHICS BOARD, UNIVERSITY OF MANITOBA (REB), approved this study. The REB had waived the requirement for informed consent in this retrospective chart review study.

Manitoba is a central Canadian province with a population of 1.2 million. Health care is publicly funded and non-participation in the plan is rare. The Manitoba Cancer Registry (MCR) is population based and a member of the North American Association of Central Cancer Registries (NAACCR), which administers a program that reviews member registries for their ability to produce complete, accurate, and timely data. Date of birth, gender, date of diagnosis, diagnosis and stage at presentation are included fields. Non-Hodgkin’s lymphoma cases diagnosed up to and including 2001 were coded using the International Classification of Diseases 9^th^ Clinical Modification ICD-9-CM and cases diagnosed after 2001 were coded using the International Classification of Diseases 10^th^ Revision, Canada. Data was collected from the paper charts and the radiation record and verify system at Cancer Care Manitoba (CCMB) Medical Record department.

Ann Arbor stage [7] was based on clinical information, imaging including CT, bone marrow biopsy (39/40) and in some cases lymphangiography. None of the patients underwent staging laparotomy or PET scan. Laboratory data including complete blood count and LDH levels were collected. Radiation treatment details were obtained from patient clinical records. All patients were evaluated for treatment response clinically and or radiographically. Recurrences were detected clinically and/or radiographically but without a standard mandated schedule of clinical follow up.

Definitions of endpoints were taken from the International Workshop to Standardize Response Criteria for Non-Hodgkin’s Lymphoma [8]. OS includes deaths from all causes. PFS includes as events recurrence if complete response (CR) or death with lymphoma but does not include death from other causes. Others have used the term freedom from treatment failure (FFTF) to indicate this measure. [9]. It should be noted that this definition of PFS differs substantially from the definition of PFS suggested by RECIST version 1.1 [10], [11] which does include death from any cause. Event free survival (EFS) [8] includes recurrence, progression and death from any cause. Times from treatment rather than from diagnosis were used because of the variable period of observation that may occur after diagnosis in largely asymptomatic patients with limited stage low grade FL.

Three hundred and twenty two patients with suspected follicular B-cell lymphoma seen between 1982 and 2008 were identified from the MCR. Patients treated initially with chemotherapy or both modalities as initial treatment were excluded. This resulted in the identification of 62 patients with stage I–II FL treated initially by radiation alone. The original histological diagnosis and grade were verified from archival slides by an experienced hematopathologist. This process confirmed 38 cases of grade I or II FL WHO classification. An additional 2 cases had been reviewed previously and classified as low grade. In 2 cases the original diagnosis was made by flow cytometry and the cases were included based on stage. The remaining 22 patients were excluded because of a grade higher than II or a disagreement about histological diagnosis.

The Follicular Lymphoma International Prognostic Index (FLIPI) [12], [13] was calculated for all patients. Patients were treated with either involved field (IF) or extended field (EF) radiotherapy. IF was defined as a field encompassing the involved disease, plus a margin including up to one adjacent nodal group proximally and distally. EF was defined as more than that, typically traditional mantle fields in supra-diaphragmatic disease or inverted-Y field in sub-diaphragmatic disease. In one case the EF consisted of treatment of the nasopharynx, oropharynx and bilateral neck for a tonsillar tumour.

Statistical analysis was done with SAS™ version 9.1. Multivariable analysis was not feasible with this number of patients and events [14].

## Results

Characteristics and univariate outcomes for the 40 patients are shown in Table 1. The mean age at diagnosis was 61.3 years (32–87). There were 26 male and 14 females. Ann Arbor stage was I in 26 and II in 14. No patient had ‘B’ symptoms. Final pathology review showed grade I FL in 21 and grade II FL in 19. A majority of patients were treated with IF radiotherapy to a mean dose of 35 Gy (15–48 Gy). The median size of the largest lymph node was 3 cm (1–9 cm). Performance status was ECOG 0 for 39 patients (97.5%) and not recorded in 1 case. Only 3 of 32 patients had an LDH value greater than normal.

**Table 1:** Patient, tumour and treatment characteristics, and univariate outcomes.

Predictor	Value	N (%)	OS	PFS	EVS
Sex	Male	26 (65)	0.57 (0.20–1.66) P = 0.30	3.97 (0.89–17.7) P = 0.072	1.65 (0.68–3.97) P = 0.27
	Female	14 (35)	ref	ref	ref
Age at Diagnosis	<60	18 (45)	ref	ref	ref
	60+	22 (55)	**4.31 (1.17–15.9) P = 0.029**	0.54 (0.19–1.56) P = 0.25	1.52 (0.65–3.57) P = 0.34
Ann Arbor	I	26 (65)	2.22 (0.68–7.24) P = 0.19	0.49 (0.17–1.41) P = 0.19	0.96 (0.41–2.26) P = 0.93
	II	14 (35)	ref	ref	ref
FL Grade	1	21 (52)	ref	ref	ref
	2	19 (48)	1.22 (0.42–3.53) P = 0.71	0.8 (0.28–2.30) P = 0.68	1.05 (0.47–2.37) P = 0.90
Haemoglobin	<145	16 (43)	ref	ref	ref
(3 missing)	145+	21 (57)	0.30 (0.08–1.12) P = 0.072	1.73 (0.58–5.21) P = 0.33	1.07 (0.46–2.5) P = 0.87
RT Dose	< = 35	26 (65)	ref	ref	ref
	>35	14 (35)	1.32 (0.45–3.83) P = 0.61	0.59 (0.18–1.93) P = 0.39	0.73 (0.31–1.72) P = 0.48
Field	IF	36 (90)	0.86 (0.23–3.19) P = 0.82	**0.22 (0.07**–**0.71) P = 0.012**	0.33 (0.11–1.01) P = 0.053
	Other	4 (10)	ref	ref	ref
FLIPI	Low (0,1)	26(84)	**0.15 (0.04**–**0.61) P = 0.008**	not calculable	0.94 (0.31–2.86) P = 0.91
(9 missing)	Intermediate (2)	5(16)	ref		ref
	Missing values	9(23)			
Lymph Node Size	>3 cm	16	0.56 (0.19–1.70) P = 0.31	0.90 (0.31–2.59) P = 0.84	0.86 (0.38–1.97) P = 0.72
	1–3 cm	22	ref		
	missing	2			

Median follow up was 6.9 years. The median time from diagnosis to radiation treatment was 3.3 months (0.5 mo. to 5.5 years). All patients achieved a clinical CR. Gender, grade, dose, side of diaphragm; stage I vs. stage II, haemoglobin and size of the lymph node were not prognostic for PFS or OS. The FLIPI was low risk (0–1 adverse factor) in 26 patients and intermediate risk (2 adverse factors) in 5 patients. None of the patients with FLIPI score 2 recurred. There were 14 patients (crude rate 35%) with recurrent disease after radiation therapy of whom 5 died. Only 8% (3 of 40 patients) failed within the radiation field giving a crude rate of 92% for local control. Overall 24 subjects died or had a recurrence, and 10 died without recurrence.

**Figure 1:**
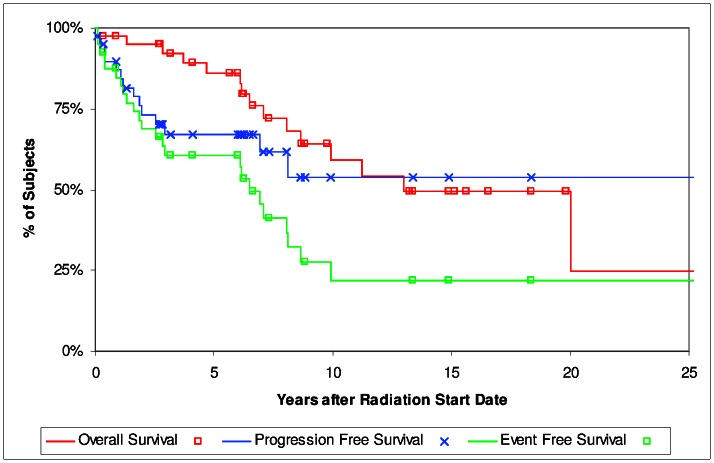
Overall survival, progression free survival and event free survival for all patients.

Figure 1 shows PFS, OS and EFS for all patients. The median OS post-radiation was 13.0 years, median EFS was 6.5 years. Five and 10 year OS for all patients was 86% and 59%, respectively. The 5 and 10 year PFS were 67% and 54%. The 5 and 10 year EFS for all patients was 60% and 22%. Patients ≥60 years old were at a higher risk of dying, HR 4.31, (95% CI 1.17–15.9) *p* = 0.030 but this includes all causes. Age was not a prognostic factor for EFS, HR 1.52 (95% 0.65–3.57) *p* = 0.34.

After first relapse, patients received chemotherapy, radiation, both or observation. 9 out of the 14 patients with relapse obtained a second clinical CR and were alive and disease free at last follow up.

## Discussion

Our PFS of 54% and OS of 59% at 10 years and 92% local control are consistent with previous radiation-only series published within the last 15 years [5], [6], [9], [15]–[26]. In these series stage I and II FL treated with RT alone produces PFS of 30–50%, OS of 60–70% at 10 years and local control in the range of 90–95%. Our definition of PFS which censors death other than lymphoma is consistent with the published data. We also report EFS of 22% at 10 years. This outcome measure includes recurrence, progression and death from any cause. Authors recognize that there are only 40 patients analyzed in this study with a median follow up of 6.9 years. This makes it challenging to interpret data in a malignancy which has an indolent course.

Despite advances in imaging, pathological classification and treatment technology these results are similar to those reported 25 years ago by Gospodarowicz [27]. In a recently reported Surveillance, Epidemiology, and End Results (SEER) study that included 6568 patients with early stage follicular lymphoma, 34% (2222) were initially treated with radiotherapy. Disease Specific Survival (DSS) and OS for RT and no RT groups were 79% VS 65%, (P<.0001) and 62% vs. 48% (P<.0001) [28]. Observation alone is a reasonable option when potential toxicity from radiation out weighs its benefit because of the extent and location of the disease [4]. In the pre-rituximab era chemotherapy had not been convincingly shown to improve treatment results compared to radiation alone [5]. An analysis of 144 stage 1 (by bone marrow) FL patients grade I–II enrolled in the National Lymphocare data base with a median follow up of 57 months reported that patients who received either rituximab and chemotherapy (R-chemo) or R-chemo plus radiotherapy had significantly improved PFS but no difference in OS compared with patients receiving radiotherapy alone. [29] The combination of RT and rituximab is currently under investigation by the German Low Grade Lymphoma Study Group (NCT00509184) [30].

A plateau in recurrences has been noted by some [6] after 10 years suggesting a number of patients so treated might be cured and our data are certainly consistent with this. However in the absence of a control group there is no class I evidence that radiation therapy has any impact on survival in limited stage FL. The long-term survival of some patients may be a feature of the disease, and not a result of the treatment. [4].

A randomized trial is underway in Germany including limited stage FL comparing extended field and total nodal irradiation [31]. If this trial shows a difference in OS then we may have some information about the impact of treatment on overall outcome. A study of 24 conventionally staged patients with stage I–II FL treated with involved field radiation, showed sustained molecular response of peripheral blood and bone marrow Bcl-2/IgH+ cells found by PCR at diagnosis in 9 of 15 patients for a median of 43.5 months. Relapse was more common in those who never cleared circulating Bcl-2/IgH+ cells compared to those who did clear or had a negative baseline test [32].This study provides indirect evidence that local treatment could reduce systemic risk and cure some patients.

Age was prognostic for overall survival (OS), but because of the age of this population and competing mortality, this is not an end point that measures treatment effectiveness well. Therefore we have also examined progression free survival (PFS) which censors those who have died from another cause without prior FL recurrence [8] and provides a signal of treatment effectiveness for disease control with the noise of other illnesses removed. PFS may be a preferred end point when many deaths are unrelated to cancer as is the case in this older population. However, this supposes that death certificates are accurate, and that lymphoma was not present at time of death. Because FL could in fact be present but not recorded on a death certificate, and thereby an antecedent recurrence not recorded, PFS represents a maximum estimate of treatment effectiveness. On the other hand Event Free Survival (EFS) which includes recurrences and death from all causes as events indicates those who are both alive and free of disease and provides a measure of efficiency of treatment. Because of progressive disease plus unrelated deaths, the EFS at 10 years was only 22%.

The FLIPI which includes age, stage, hemoglobin, LDH and number of nodal sites was prognostic for OS in this homogeneous group of selected patients treated with radiation. All relapses occurred in low risk (score 0–1) patients, and none of the intermediate risk patients (score 2) relapsed. Only 4 had low hemoglobin, 3 had elevated LDH, and none had bulky disease. These “reversed” results make it very unlikely that FLIPI would be prognostic with such selected patients even with a larger sample size. This is in contrast to the results of Plancarte [13] where FLIPI was prognostic in stage I and II FL, but that series included grade III cases and also bulky disease. These authors suggested that pre-treatment case selection may improve results and our series, which excluded grade III FL and had only 4 with intermediate risk FLIPI may indicate such homogeneity indeed renders the FLIPI non-discriminant. Other factors that have been prognostic in other series include B symptoms and bulk. None of our patients had B symptoms and all had nodes ≤9 cm. Therefore it is perhaps not surprising that we have not found any prognostic factor beyond age, with the caveat of sample size and β error.

Dose greater or less than median (35 Gy) were not found to be predictive of OS or PFS suggesting that ≤35 Gy may be sufficient for the control of this disease [5]. In a randomized trial, which compared 40–45 Gy vs. 24 Gy, and included 169 patients with grade I–II early stage FL, there was no difference in any outcome. [33].

### Conclusions

In a group of patients carefully selected for stage I or II pathologically verified WHO grade I or II FL and treated with radiation alone, CR after a median 35 Gy was 100% and local control was 92%. The longer-term outcomes for PFS, OS and local control from this single institution study are consistent with previously published reports. Age, other clinical factors, treatment factors and FLIPI were not prognostic for PFS in this homogeneous population. We conclude radiation therapy provides definitive local control and a relapse free state for a majority of patients for several years.
